# Continuous Blue Light Treatment Enhances the Nutritional Value of Hydroponically Grown *Eruca vesicaria* L. by Improving Ascorbic Acid Biosynthesis

**DOI:** 10.3390/foods13132141

**Published:** 2024-07-05

**Authors:** Gabriele Paglialunga, Stefano Moscatello, Alberto Battistelli, Michele Mattioni, Marta Del Bianco, Simona Proietti

**Affiliations:** 1 Research Institute on Terrestrial Ecosystems, National Research Council, 05010 Porano, Italy; gabriele.paglialunga@iret.cnr.it (G.P.); alberto.battistelli@cnr.it (A.B.); michele.mattioni@cnr.it (M.M.); simona.proietti@cnr.it (S.P.); 2 Italian Space Agency, 00133 Rome, Italy; marta.delbianco@asi.it

**Keywords:** controlled-environment agriculture, light management, vitamin C, spectral composition, LEDs, crop quality

## Abstract

This study investigates the effect of continuous blue light (CBL) treatment on quality-related metabolites, focusing on ascorbic acid (AsA) accumulation in hydroponically grown *Eruca vesicaria* (L.). Plants were subjected to CBL treatment, consisting of 24-h exposure to constant-intensity blue light (48 μmol m^−2^ s^−1^) and 12-h exposure to the remaining spectrum (192 μmol m^−2^ s^−1^). The activities of key enzymes in AsA biosynthesis and recycling were analyzed, including L-galactono-1,4-lactone dehydrogenase (GalLDh), monodehydroascorbate reductase (MDhAR), dehydroascorbate reductase (DhAR), and ascorbate peroxidase (APX). The results showed a significant increase in AsA accumulation of 65.9% during the “day” and 69.1% during the “night” phases under CBL compared to controls. GalLDh activity increased by 20% during the “day phase” in CBL-treated plants. APX activity also rose significantly under CBL conditions, by 101% during the “day” and 75.6% during the “night”. However, this did not affect dehydroascorbic acid levels or the activities of MDhAR and DhAR. These findings highlight the potential of tailored light treatments to enhance the nutraceutical content of horticultural species, offering valuable insights for sustainably improving food quality in controlled-environment agriculture (CEA) systems and understanding the roles of blue light in ascorbic acid biosynthesis.

## 1. Introduction

The introduction of LED technology has marked a revolutionary shift in agricultural practices, providing growers and researchers with unprecedented precision in adjusting light parameters toward better control over crop production [1,2]. Notably, LEDs, distinguished by their adaptable spectrum and energy efficiency, have risen to prominence as the preferred lighting technology in controlled-environment agriculture (CEA) [3,4]. Scientists have undertaken thorough investigations to examine the impacts of diverse light treatments on plant production and quality response. These studies have delved into a wide range of light management, encompassing different light intensity levels [5,6], modifications in light spectral composition [7,8], alterations in photoperiod durations [9,10], pre-harvest supplemental treatments [11,12], and post-harvest interventions during storage [13,14].

Continuous light (CL) treatment, characterized by exposure to continuous illumination without a normal day–night rhythm, has been the subject of extensive scientific investigation in the context of crop production [15]. This approach is based on the concept of diminishing light intensity while extending the duration of illumination over 24 h, aiming to achieve a substantial daily light integral (DLI) while concurrently reducing the number of photons delivered per unit of time [9]. Scientific studies have explored the impact of CL on plant biomass accumulation, photosynthetic efficiency, and nutritional quality, demonstrating its potential to influence key physiological processes [15,16,17]. Apart from assessing the physiological response of plants to CL to quantify tangible gains in productivity and quality in CEA, other potential benefits encompass resource efficiency, including a reduction in the electric power peaks demanded by the system, as well as a decrease in the size requirements of the illumination and cooling systems [9].

Beyond CL, research exploring the effects of specific light spectra on plant physiology has generated substantial insights into the multifaceted ways light shapes crop production and quality [18,19,20]. Notably, studies have demonstrated that blue light might play a relevant role in modulating plant metabolisms and subsequent various quality-related aspects such as antioxidant capacities in several plant species [21,22,23,24]. Therefore, the ongoing exploration of blue light management in CEA on a species-specific basis holds the potential to provide valuable insights for improving the nutraceutical content and quality of horticultural species.

Commonly referred to as Vitamin C, ascorbic acid (AsA) stands out as a widely recognized antioxidant and one of the most prevalent vitamin supplements used globally. Adequate levels of AsA are crucial for maintaining optimal physiological function in the human body [25]. Vegetables serve as the primary source of this essential compound, which plays a significant role in defining the nutritional value of foods. Interestingly, AsA content has recently been regarded as one of the primary quality-driving attributes of vegetable-based foods in the context of human space exploration as a potential diet component that might play an important role in radiomitigation strategies [26,27,28].

Among various studies, it has been demonstrated that the pool of AsA in horticultural species can be regulated by the blue light component of the spectrum. Specifically low red–blue light ratios increase the total ascorbic acid content in lettuce [29]. Another study demonstrated that blue light radiation enhances AsA accumulation in Chinese cabbage seedlings by activating gene expression involved in the biosynthetic and recycling pathways [30]. End-of-production treatment with a high proportion of blue light irradiation improved AsA accumulation by enhancing the activities of key enzymes responsible for AsA synthesis, improving the nutritional quality of lettuce [31].

The most effective exogenous precursor of AsA is L-galactono-1,4-lactone, which is directly converted to AsA by the mitochondrial enzyme L-galactono-lactone-1,4-lactone dehydrogenase (GalLDh). The AsA pool is also regulated through the AsA recycling pathway, involving the enzymatic activity of monodehydroascorbate reductase (MDhAR) catalyzing the reduction of monodehydroascorbic acid (MDhA) in AsA, dehydroascorbate reductase (DhAR) catalyzing the reduction of dehydroascorbic acid (DhAs) in AsA, and ascorbate peroxidase (APX), which is responsible for AsA oxidation in MDhA and can spontaneously dissociate to DhAs. Manipulating the blue light spectrum alongside prolonged photoperiods represents a promising strategy for augmenting AsA content in responsive plant species.

*Eruca vesicaria* (L.), a member of the Brassicaceae family, stands out as a food crop with considerable importance, given its substantial commercial value, especially as a widely favored ingredient in fresh salads around the world. Moreover, it demonstrates the capability to thrive and yield in CEA [32,33], as well as great performance under CL conditions [9].

Given the need and opportunity to enhance produce quality and improve light use efficiency in CEA, in this study, we chose to implement a CL treatment on hydroponically grown *Eruca vesicaria* L., specifically focusing on the blue wavelength. This approach aimed to provide 24 h of constant-intensity blue light (48 PPFD) and 12 h of full-spectrum light (192 PPFD) only during the “day phase”. The first aim was to verify the hypothesis that continuous blue light might elicit an increase in AsA in *E. veiscaria*, as reported in the scientific literature for several other plant tissues and species grown under higher absolute and relative intensities of blue light. This would allow an increase in quality with no increase in energy expenditure. Additionally, in order to study the biochemical control of the AsA pool size, we assayed the activity of key enzymes of the main biosynthetic and recycling pathways including GalLDh, MDhAR, DhAR, and APX.

## 2. Materials and Methods

### 2.1. Plant Materials and Growth Conditions

Rocket, *Eruca vesicaria* (L.) Cav., seeds were provided by “La Semiorto Sementi” (Sarno Salerno, Italy). Plants were grown at the Consiglio Nazionale delle Ricerche-Istituto di Ricerca sugli Ecosistemi Terrestri (CNR-IRET) under fully controlled environmental conditions in a growth chamber (Fitotron SGD170 Sanyo; Gallenkamp, Leicestershire, UK). The chamber, which was equipped with two LED lamps (model LX60; Heliospectra AB, Gothenburg, Sweden), was divided into two sections (one for treatment and one for control) by a reflective and perforated panel, allowing air circulation and maintaining a homogeneous environment in both sections. The LED lamps were equipped with four types of dimmable (0–1000) LEDs. The emission characteristics of the different LED types were as follows: blue LEDs (B), 450 nm; red LEDs (R), 660 nm; far-red LEDs (FR), 730 nm; and cool white LEDs (W), 5700 K. The W LEDs had an emission spectrum of B = 26%, green (G) = 45%, R = 27%, and FR = 2%.

Two seeds were sown in a 2 mL Eppendorf tube (Eppendorf Srl, Milan, Italy), whose bottom end was removed and filled with perlite and vermiculite. The Eppendorf tubes were positioned in plastic racks (24 × 34 cm) each with 224 seats, leaving a row of empty seats between each row of Eppendorf tubes, covering a total of 112 seats. Each rack was fixed on the top of a container with a water capacity of eight liters. Two containers per treatment were used in the experiment.

Seeds were germinated under a 150 μmol m^−2^ s^−1^ photosynthetically photon flux density (PPFD), provided for 12 h per day (12-h dark) by cool white 5700 K LEDs at 25 °C with 90% relative humidity for nine days after emergence (DAE), which is when the treatment started (time zero). At this stage, 112 plants were grown in each container (plant sowing density of 2105 plants m^−2^) and the water was substituted with an aerated and complete nutrient solution such as the one used in [9]. The composition of the nutrient solution was as follows: NH_4_ 0.122 mol, K 1.028 mol, Ca 0.419 mol, Mg 0.135 mol, NO_3_ 1.785 mol, Cl 0.068 mol, SO_4_ 0.109 mol, P 0.189 mol, Fe 3.795 mmol, Mn 0.189 mmol, Zn 0.244 mmol, B 2.840 mmol, Cu 0.068 mmol, and Mo 0.042 mmol. The nutrient solution was renewed every week, ensuring that pH and EC were maintained at 6.5 and 1.7 mS cm^−1^, without relevant changes. A temperature of 25 ± 1 °C/22 ± 1 °C day/night, relative air humidity of 70 ± 1%, and CO_2_ partial pressure of 400 ppm were maintained.

### 2.2. Light Treatment

**Figure 1:**
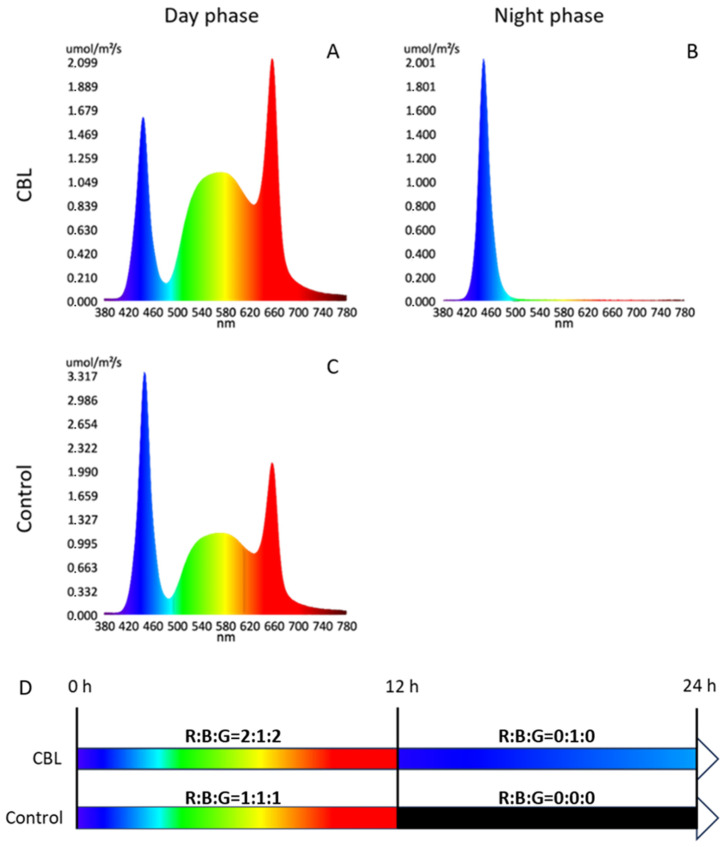
Spectrum detected in the various treatments. In the upper left quadrant, (**A**) continuous blue light (CBL) treatment during the “day phase” with red (96 μmol m^−2^ s^−1^), blue (48 μmol m^−2^ s^−1^), and green (96 μmol m^−2^ s^−1^) spectral bandwidths; in the upper right quadrant, (**B**) CBL treatment during the “night phase” with only blue (48 μmol m^−2^ s^−1^) spectral bandwidth; in the lower left quadrant, (**C**) control during the “day phase” with red (96 μmol m^−2^ s^−1^), blue (96 μmol m^−2^ s^−1^), and green (96 μmol m^−2^ s^−1^) bandwidths; no spectrum of the control during the “night phase” is presented as light was not applied during this phase; (**D**) schematic diagram of the light setup.

**Figure 2:**
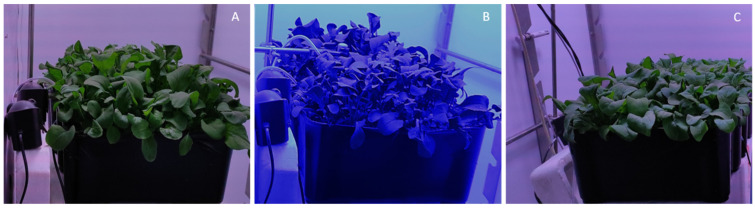
Images of *Eruca vesicaria* during (**A**) continuous blue light (CBL) treatment during the “day phase”; (**B**) CBL treatment during the “night phase”; (**C**) control during the “day phase”.

The following light treatments started from time zero and lasted until harvest: (1) Control with a photoperiod of 12-h light/12-h dark and PPFD of 288 μmol m^−2^ s^−1^ provided by W, R, and B LEDs to obtain a final R–B–G ratio of 1:1:1 and a total of 96 μmol m^−2^ s^−1^ of blue light (Figure 1C and Figure 2C); (2) continuous blue light (CBL) treatment with a photoperiod of 24-h that provides a continuum of 48 μmol m^−2^ s^−1^ of blue light (Figure 1A,B and Figure 2A,B) and 12 h of the rest of the spectrum at 192 μmol m^−2^ s^−1^ provided by W, R, and B LEDs with an R–B–G ratio of 2:1:2 (Figure 1A). Both light treatments received the same daily cumulative intensity of photosynthetically active quanta (as daily light integral—DLI = 12.44 mol m^−2^ day^−1^).

For simplicity through the text, the 12 h at 48/0 (CBL/Control) μmol m^−2^ s^−1^ of blue light is defined as the “night phase” while the 12 h at 240/288 (CBL/Control) μmol m^−2^ s^−1^ of the full spectrum is defined as the “day phase”. The emission of light, encompassing both the intensity and spectral composition across different treatments, was measured by an LI-180 spectrometer (LI-COR inc., Nebraska, NE, USA).

### 2.3. Plant Measurements and Compositional Analysis

Harvest occurred 20 days after time zero. Plants in each rack were divided into 4 samples (eight replicates per treatment), for each of which fresh weight, dry weight, dry matter percentage, the leaf area index (LAI), and ash content were determined.

Leaves were freeze-dried until reaching a constant weight to determine the dry weight. For the calculation of LAI, the leaf area of four sub-samples of 20 g of fresh leaves for each growing tank was measured by ImageJ software (version 1.54i―an open-source image processing software) [34]. The LAI was then calculated on the basis of the total fresh weight of leaves per unit of growing area. The specific leaf dry weight (SLDW) was calculated using four leaf discs of 1.90 cm^2^ for each rack (8 leaf discs for each treatment) cut from the lamina with a cork borer.

The ash content was only determined for the shoot via the ignition of the dried powder at 575 °C in a furnace (P 300; Nabertherm GmbH, Lilienthal, Germany), with the temperature increased according to the NREL/TP-510-42622 method.

For the quantification of non-structural carbohydrates (NSCs), pigments, total phenolic content, total anthocyanins, organic acid, and inorganic anion concentration, fresh samples were collected before the harvest at the end of the “night phase” and immediately frozen in liquid nitrogen, ground into a fine powder in a mortar using a pestle under liquid nitrogen conditions, and preserved at −80 °C until analyses.

For AsA analysis and AsA-related enzyme activities, plants were sampled at harvest at two times during the photoperiod: (1) at the end of the “night phase” and (2) at the end of the “day phase”.

All the biochemical analyses, including phytochemical content and enzyme activity assays, were conducted on eight biological replicates per treatment for each parameter.

### 2.4. Ascorbic Acid Assay

For the determination of total ascorbic acid (Tot. AsA) content, 2 mL of 3% Metaphosphoric acid (MPA) at 4 °C was used for extraction in 30 mg of frozen powder in an ice-cold glass–glass homogenizer. The mixtures were centrifuged at 16,000× *g* for 5 min at 4 °C. The supernatants were filtrated through a 0.2 μm (Whatman) PPII nylon filter and then separated into two aliquots in HPLC vials, one for direct Tot. AsA quantification and the other for DhAs reduction.

DhAs was reduced to AsA using 5 mMol L^−1^ Tris (2-carboxyethyl) phosphine (TCEP) as a reducing agent after incubation for 30 min at 25 °C. After 30 min, the samples were injected into HPLC for the quantification of Tot. AsA.

Tot. AsA was analyzed using an UltiMate 3000 HPLC System ThermoScientific^™^ Dionex (Sunnyvale, CA, USA) coupled with a UV/VIS detector (ThermoScientific^™^ Dionex). The separation was performed using a Phenomenex Luna C18 column (250 mm × 4.6 mm i.d. and particle size 5 μm) and the run time was 15 min. The Tot. AsA peak was detected at 254 nm and the processing of the chromatographic peaks was performed using the software version Chromeleon 7.2 (ThermoScientific^™^ Dionex). The entire chromatographic separation was performed in an isocratic mobile phase consisting of 0.010 mol L^−1^ of KH_2_PO_4_, maintained at pH 2.8 and a flow rate of 0.7 mL min^−1^ with an injection volume of 5 μL. Quantification was performed by means of a calibration curve of an AsA standard.

All the reagents used are of a high degree of purity for HPLC analysis. The Tot. AsA content was expressed as mg 100 g^−1^ of fresh weight (FW).

### 2.5. Non-Structural Carbohydrates Assay

Measurements of non-structural carbohydrates (NSCs) were performed by extracting 10 mg of the frozen powder. The extraction was performed in 1 mL of 80% ethanol at 80 °C for 45 min under continuous shaking. Following extraction, the mixture was centrifuged at 16,000× *g* for 5 min to separate soluble sugars (glucose, fructose, and sucrose) in the supernatant from starch in the pellet.

Spectrophotometrically coupled enzymatic assays [35] were utilized for soluble sugar determination, with measurements taken in dual-wavelength mode (340–405 nm) using an Anthos plate reader (Anthos Labtec Instruments, Wals, Austria).

The pellet, containing starch, was washed four times with a 50 mM Na-acetate buffer (pH 4.5) and then suspended and autoclaved at 120 °C for 45 min in 1 mL of the same buffer. After autoclaving, the sample was incubated at 50 °C for 1 h with amyloglucosidase (70 U) and α-amylase (4 U) to hydrolyze the starch to glucose. The glucose produced by starch hydrolysis was then measured as previously described by a spectrophotometrically coupled enzymatic assay.

All the NSC contents were expressed as mg 100 g^−1^ FW.

### 2.6. Pigments Assay

Chlorophylls (*a* and *b*), β-carotene, lutein, neoxanthin, and violaxanthin were extracted from 100 mg of frozen powder using 2 mL of 100% acetone at 4 °C in the absence of light, employing a glass–glass homogenizer. Subsequently, the samples underwent centrifugation at 16,000× *g* for 5 min at 4 °C and the supernatants were then filtered through a 0.2 μm nylon PPII syringe disposable filter.

For analysis, 15 μL of the resulting clear extract was injected into an HPLC U3000 system (Dionex™ ICS-5000; Thermo Fisher Scientific, Waltham, MA, USA) fitted with a C18(2) LUNA (Phenomenex, Bologna, Italy) analytical column (5 μm, 250 mm × 4.6 mm) and a corresponding guard column (Phenomenex, Bologna, Italy) maintained at 30 °C. The separation was accomplished isocratically, with the mobile phase consisting of solution A (1.75% water, 1.75% methanol, 1.75% dichloromethane, and 94.75% acetonitrile) from 0 to 4 min and solution B (50% acetonitrile and 50% diethyl acetate) from 4.1 to 18 min, followed by a 4 min re-equilibration with solution A. The flow rate was maintained at 1 mL min^−1^, resulting in a total run time of 22 min. The autosampler temperature was set to 4 °C, and the UV detector wavelength was fixed at 440 nm.

The concentrations of all pigments were determined via comparisons with standard curves. Finally, the pigment contents were expressed as mg per 100 g of FW.

### 2.7. Total Anthocyanins and Total Phenolic Assay

Total anthocyanins (Tot. Ant) were quantified through the extraction of 100 mg of frozen powder in 2 mL of 1% HCl in methanol, followed by incubation for 1 h at 65 °C. The resultant liquid extract underwent centrifugation at 16,000× *g* for 5 min to facilitate separation. Post-centrifugation, the supernatant was isolated, and the Tot. Ant content was determined spectrophotometrically by dual-wavelength absorbance measurements at 530 nm and 657 nm. The absorbance reading at 657 nm was utilized to correct the anthocyanin quantification by accounting for potential interference from chlorophyll degradation products.

The quantification of the phenolic component (TPC) involved the extraction of 15 mg of frozen powder in 2 mL of 100% methanol. Following centrifugation at 16,000× *g* for 5 min, the supernatant was harvested for the spectrophotometric quantification of TPC, with absorbance readings recorded at 765 nm.

The determination of TPC was accomplished by establishing a linear relationship between the absorbance of each sample and the corresponding concentration of gallic acid, serving as a calibration standard.

The resulting values for Tot. Ant content and TPC were expressed as mg per 100 g of FW.

### 2.8. Anions Assay

The extraction of inorganic anions (nitrate, sulfate, and phosphate) and organic acids (malate and citrate) was performed using 100 mg of frozen powder suspended in water at 80 °C for 45 min with continuous stirring. Post-extraction, the samples were subjected to centrifugation at 16,000× *g* for 5 min, and the resultant supernatant was collected and passed through a 0.2 μm nylon PPII syringe filter before being introduced into an ion chromatography system (DionexTM ICS-5000).

The ion chromatography system featured a conductivity detector, an IonPac AS11-HC analytical column (4 × 250 mm) paired with a guard column, and an IonPac Anion Trap Column (ATC)-1 (Thermo Fisher Scientific, Waltham, MA, USA). To mitigate unwanted ion interference, an ERSTM 500 electrolytically regenerated suppressor was integrated into the chromatographic system. Operational parameters included a temperature of 30 °C and a flow rate of 1 mL min^−1^. The mobile phase comprised a gradient of 100 mM NaOH, transitioning from 1 to 15 mM over a span of 24 min. Signal detection was quantified in micro-Siemens (μS). Both eluents and standard solutions of inorganic anions were prepared using HPLC-grade reagents (Merck KGaA, Darmstadt, Germany). Chromatographic system control, along with data acquisition and processing, was achieved by Chromeleon 7.2 software (ThermoScientific™ Dionex).

The quantification of nitrate, sulfate, and phosphate was expressed as ppm while malate and citrate content were expressed as mg per 100 g of FW.

### 2.9. Ascorbic Acid Biosynthetic and Recycling Enzyme Activity Assay

Galactono 1-4 lactone dehydrogenase (GalLDh) (EC 1.3.2.3) activity was assayed by monitoring the reduction in Cytochromes c (Cyt c) at 550 nm according to [36] with some modification. 100 mg leaf tissues were extracted by 2 mL of a pre-cooled extraction buffer, which contained 100 mM PBS (pH 7.4), 0.4 M sucrose, 10% (*v/v*) glycerol, 1 mM EDTA, 5 mM TCEP, and 1% (*w/v*) polyvinylpyrrolidone (PVP). The reaction mixture (1 mL) contained 50 mM potassium phosphate (pH 7.8), 1.05 mg/mL Cyt c, 5.6 mM L-Galactono-1,4-lactone, and 0.1 mL enzyme extract. One unit of the enzyme was defined as the activity that oxidized 1 µmol of L-Galactono-1,4-lactone corresponding to the reduction in 2 µmol of Cyt c per min.

For the assay of monodehydroascorbate reductase (MDhAR) (EC 1.6.5.4) and dehydroascorbate reductase (DhAR) (EC 1.8.5.1), 100 mg of leaf tissues was homogenized in 2 mL of the pre-cooled extraction buffer. The extraction buffer contained 50 mM PBS (pH 7.5), 1 mM EDTA, 0.1% (*v/v*) Triton X-100, 0.2% (*v/v*), and 2% (*w/v*) polyvinylpyrrolidone (PVP). The extract was centrifuged at 4 °C, and then the supernatant was used for spectrophotometric measurements.

MDhAR activity was assayed by monitoring the absorbance at 340 nm. The reaction mixture (3 mL) contained 50 mM HEPES-KOH (pH 7.6), 0.5 mM ascorbic acid, 0.3 mM NADH, 0.5 units ascorbate oxidase, and 0.1 mL enzyme extract. One unit of the enzyme was defined as the amount that oxidized 1 µmol of NADH per minute.

DhAR activity was assayed by monitoring the absorbance at 265 nm. The reaction mixture (3 mL) contained 100 mM HEPES-KOH (pH 7.0), 1 mM EDTA, 2.5 mM GSH, 0.6 mM DhAs, and 0.1 mL of the enzyme extract. One unit of the enzyme was defined as the amount that reduced 1 µmol of DhAs per min.

To assay Ascorbate peroxidase (APX) (EC 1.11.1.11) activity, 100 mg of leaf tissues was extracted by the same extraction buffer used for GalLDh. The enzyme activity was determined by monitoring the reduction in ascorbic acid absorbance at 290 nm. The reaction mixture (3 mL) contained 50 mM potassium phosphate (pH 7.8), 0.5 mM AsA, 0.25 mM H_2_O_2_, and 0.1 mL of enzyme extract. The reaction was triggered by adding H_2_O_2_. One unit of the enzyme was defined as the amount that oxidized 1 µmol of AsA per min.

### 2.10. Statistical Analysis

The statistical analysis was carried out using IBM SPSS Statistics 20 (Chicago, IL, USA). All the parameters of the experiment were subjected to a one-way analysis of variance (ANOVA) with the Control and CBL treatment as factors. Additionally, for Tot. AsA, AsA, DhAs, and enzyme activities (GalLDh, MDhAR, DhAR, and APX), where samples were collected at two different times, ANOVA was performed using the “day phase” and the “night phase” as factors in order to individuate potential differences between the two phases within each treatment.

## 3. Results

### 3.1. Biomass Assay

A statistically significant increase of 9.9% in dry matter content was observed in the CBL treatment group compared to the Control group (Table 1). Furthermore, the SLDW of vegetables treated with CBL exhibited a significant increase of 19.9% compared to the untreated samples. No significant differences in fresh weight, dry weight, ash content, and LAI between the CBL-treated group and the Control group were recorded.

### 3.2. Ascorbic Acid Content

**Figure 3:**
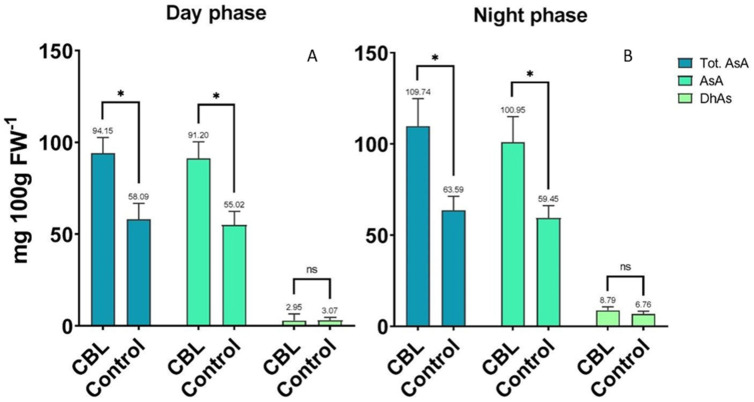
Analysis of variance and mean comparison for total ascorbic acid (Tot. AsA), ascorbic acid (AsA), and dehydroascorbic acid (DhAs) content of *Eruca vesicaria* L. samples grown under CBL and Control conditions, collected at the end of the “day phase” (**A**) and “night phase” (**B**). * Significant for *p* < 0.05, ns = not significant.

The analysis of Tot. AsA, AsA, and DhAs in the CBL group compared to the Control, sampled during both the “day phase” and the “night phase”, is summarized in Figure 3A,B. In the “day phase” samples, the CBL-treated group exhibited a significant increase in both AsA and Tot. AsA levels compared to the Control group. Specifically, there was a 65.9% increase in AsA and a 62.2% increase in Tot. AsA. In the “night phase” samples, the trend was similar, with the CBL group exhibiting a significant increase in both AsA and Tot. AsA levels compared to the Control group, with increases of approximately 69.1% and 72.5%, respectively. However, there was no significant difference in DhAs levels between the two groups. No significant difference was observed in the content of AsA, Tot. AsA, and DhAs between the “night phase” and “day phase” samples within each treatment.

### 3.3. Non-Structural Carbohydrates Content

The results of the NSC analysis are shown in Table 2. The analysis revealed no significant differences in the levels of glucose, fructose, sucrose, starch, total soluble sugars, or total non-structural carbohydrates between the CBL-treated group and the Control.

### 3.4. Pigments, Total Anthocyanin, and Total Phenolic Content

The pigment analysis revealed that chlorophyll *a*, chlorophyll *b*, total chlorophylls, and lutein showed significant increases in the CBL treatment group of 19.5%, 18.3%, 19.1%, and 18.6%, respectively, compared to the Control group (Table 3). The Chl*a*/Chl*b* ratio, β-carotene, Neoxanthin, and Violaxanthin contents did not exhibit significant variations between the groups. The results for Tot. Ant and TPC show that no significant differences were observed for Tot. Ant and TPC between the CBL-treated group and the Control group (Table 3).

### 3.5. Inorganic Anions and Organic Acids Content

The statistical analysis indicated a notable increase in malate content and a decrease in citrate in the CBL samples (Table 4). Specifically, malate demonstrated a 106.1% increase, while citrate showed a decrement of 42.74% in the CBL treatment group compared to the Control group. No significant differences in nitrate, sulfate, and phosphate levels were detected.

### 3.6. Ascorbic Acid Biosynthetic and Recycling Enzyme Activity

**Figure 4:**
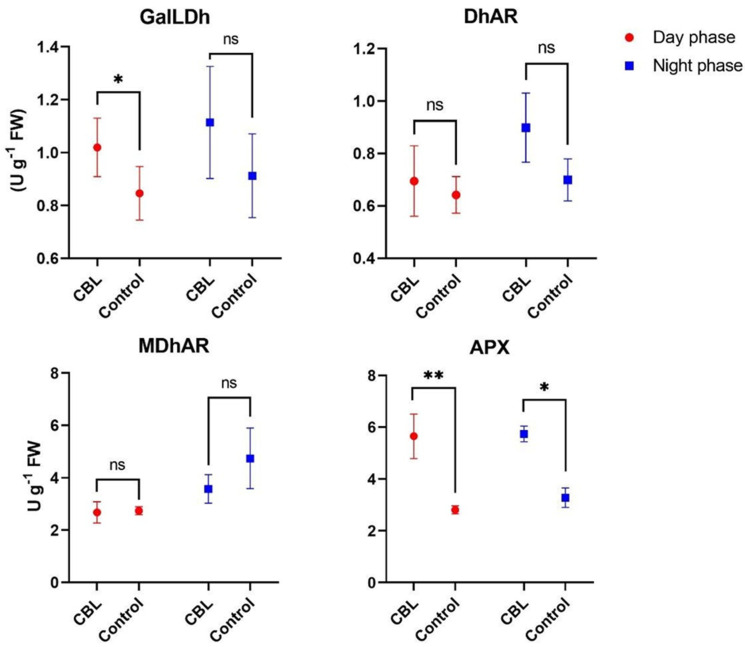
Analysis of variance and mean comparison for enzyme activity of galactono 1-4 lactone dehydrogenase (GalLDh), dehydroascorbate reductase (DhAR), monodehydroascorbate reductase, (MDhAR), ascorbate peroxidase (APX) in *Eruca vesicaria* L. samples grown under CBL and Control, collected during “day phase” and the “night phase”. *, ** Significant for *p* < 0.05 and 0.01, ns = not significant.

The enzyme activity assay results at the end of the “night phase” revealed non-significant differences in GalLDh, DhAR, and MDhAR activities between the CBL group and the Control group (Figure 4). In contrast, APX activity showed a significant increase, indicating a 75.6% increase in the treated group compared to the Control. Samples collected at the end of the “day phase” revealed a significant increase of approximately 20% in GalLDh activity in the CBL samples compared to the Control. DhAR and MDhAR activities showed non-significant differences. Notably, even during the “day phase”, APX activity exhibited a significant increase, indicating an approximately 101% rise in the CBL group compared to the Control. In the comparison between the “night phase” and the “day phase”, no significant differences were observed for any of the analyzed enzymes, neither in the CBL treatments nor in the Control group.

## 4. Discussion

In CEA conditions, environmental control can be used to modulate the productivity and quality of the produce while optimizing resource use efficiency. Light is a key factor in CEA and represents one of the most relevant costs for the facilities, affecting its economic profitability and environmental footprint [37].

Considering the significant role of Vitamin C as a fundamental nutraceutical to enhance the quality of horticultural produce, the possibility of managing light aimed at increasing the AsA content becomes promising [30,31,38,39]. A useful benchmark for ascorbic acid production efficiency could be the amount of produce providing the EFSA recommended daily dose (90 mg day^−1^ to an adult person) [40]. Under our continuous blue light treatment, rocket reveals a significant increase in Tot. AsA content compared to the controls, during both the night and day phases. With the CBL treatment, ~93 g of fresh rocket is needed to meet the daily intake of 90 mg of vitamin C, while under control conditions, ~157 g would be required. To better convey the idea of the gain achieved in terms of ascorbic acid, we obtained similar levels to those achieved in a previous study conducted by the same research team on rocket under continuous light, although in that case, the DLI was 25.92 mol quanta m^2^ day^−1^ with constant PPFD under white LED or red+blue LED [9]. As a protector against photooxidative damage in plants, the accumulation of AsA is recognized to be proportional to the level of light intensity [41]. The ability to selectively increase its content by adjusting the time of irradiation distribution of specific spectral bandwidths at low-intensity levels represents an easy and cost-saving method compared to an approach using a general increase in light intensity. In our experiment, remarkably, the objective of increasing AsA content by modulating the blue light distribution on a daily basis was achieved without undesirable effects on productivity or other analyzed variables.

The literature presents conflicting findings concerning the impact of continuous light on plant productivity [15]. While different authors demonstrated that continuous light treatments, equivalent to the control’s daily light integral, have shown increased shoot fresh and dry biomass of leafy vegetables in CEA [9,29], others have reported detrimental effects including reduced growth or leaf chlorosis [42,43,44]. This variability can be attributed primarily to species-specific responses of plants and variations in experimental conditions including the light intensity, light spectrum, duration of the treatments, and factors influencing growth beyond light exposure. Our experiment represents a new approach regarding the CL for two main reasons: (1) the discontinuity in terms of light intensities during the treatments (PPFD dropped from 240 µmol m^2^ min^−1^ to 48 µmol m^2^ min^−1^ after 12 h and vice versa); and (2) the discontinuity in terms of the light spectrum during the treatment (shifting from full spectrum to selected blue bandwidths after 12 h and vice versa).

It is well known that photosynthetic efficiency and plant growth are regulated by the synergistic effect of multiple light wavelengths, with red and blue light being particularly crucial for photosynthesis [45]. Red light comprises photons of lower energy, readily utilized by plants during photosynthesis, perfectly fitting with the absorption peak of chlorophylls [46]. Blue light, in addition to imparting energy to photosystems, also promotes photosynthesis by stimulating stomatal opening, allowing higher CO_2_ availability at the carboxylation sites [47]. In our experiment, the diminished intensity of blue light imposed during the “day phase” in the CBL treatment did not reduce fresh and dry weight compared to the control; in contrast, it slightly increased the dry matter percentage and SLDW.

Moreover, in our experiment, a significant increase in chlorophyll and lutein content was observed. Findings related to the effect of blue light on pigment concentrations are present in the literature [18,48]. Blue light was observed to be beneficial for the accumulation of chlorophylls and carotenoids in comparison to red light [49]. It was shown that blue light could reverse red-light-induced pigment inhibition responses [50]. Moreover, chlorophyll and carotenoid content per unit of leaf area increased with the percentage of blue light up to 50% [51]. Long-term blue light treatment promoted the accumulation of lutein and β-carotene in Chinese cabbage [52]. On the other hand, although various studies demonstrated the influence of blue light on the accumulation of polyphenols and anthocyanins [53,54], in our work, we did not find significant differences, attributable to the treatment, for these compounds.

Non-structural carbohydrates represent the photosynthetic end product at the leaf level and a substantial portion of the total dry matter (approximately 25%), yet our study did not detect significant differences in glucose, fructose, sucrose, or starch. This finding indicates that non-structural carbohydrates did not contribute to the observed increase in the percentage of dry matter or SLDW in samples treated with CBL. This also indicates that photosynthetic efficiency was not altered due to the treatment, supporting the hypothesis that the higher AsA content was not a result of increased substrate availability due to improved photosynthesis. Moreover, the total LAI was not affected by the blue light modulation treatment, and this evidence is in contrast with the hypothesis of reduced cell expansion in treated plants, causing an increase in AsA simply as a result of greater tissue compactness due to the blue light effect [49,55,56,57].

Consistent with the increase in AsA in plants under our CBL conditions, there was also an enhancement in the enzymatic activity of GalLDh in samples collected during the day. The enzyme catalyzes the final step of ascorbic acid biosynthesis, and several authors confirmed its role in increasing the ascorbic acid pool in response to light stimulation, both in terms of intensity and photoperiod [36,41,58,59]. The role of light spectrum quality on the accumulation and biosynthesis of ascorbic acid has been previously assessed in lettuce with spectrum ratios under continuous light set at 75R–25B, 50R–50B, and 25R–75B, all with a light intensity of 200 μmol m^−2^ s^−1^ [38]. Higher relative blue light intensity (25R–75B) led to an increase in the total ascorbic acid content, primarily attributed to higher activity of recycling enzymes such as MDhAR and DhAR rather than increased activity in the main biosynthetic pathway [38]. However, in the latter study, even though the total ascorbic acid was significantly increased, the content of ascorbic acid and dehydroascorbic acid did not differ among the various treatments [38]. In contrast, in a recently published work, blue light promoted the accumulation of ascorbic acid in tomato fruit through the deactivation of PAS/LOV photoreceptors that inhibit the activity of GGP (GDP-L-galactose phosphorylase), which is known as a crucial regulatory step in the main biosynthetic pathway of ascorbic acid [60].

In our study, APX activity significantly increased in continuous light treatments with blue light, but in contrast, the dehydroascorbic acid content did not differ significantly either among the treatments or between night and day. These data suggest that there is proportional ascorbic acid recycling activity to counteract oxidation due to APX activity in CBL, which, in our case, has efficiency in ascorbic acid consumption equal to 99.3 mg min^−1^ on a 100 g basis. However, the analysis of the enzymatic activities of MDhAR and DhAR showed no significant differences between the blue light treatment and the control.

It is known that monodehydroascorbic acid can be spontaneously reduced to ascorbic acid by ferredoxin present in chloroplasts. Monodehydroascorbic acid is reduced in the thylakoid membranes of spinach by ferredoxin, and the contribution of MDhAR to ascorbic acid recycling could be minimal or even negligible [61]. Furthermore, it has been hypothesized that in chloroplasts, dehydroascorbic acid is not accumulated due to the activity of MDhAR and ferredoxin [62]. The latter spontaneously reduces monodehydroascorbic acid in illuminated chloroplasts without the contribution of enzymes and is then regenerated by Ferredoxin-NADP+ reductase. In our work, the hypothesis is that ferredoxin might be sufficient to “counteract” the high activity of APX in plants exposed to continuous blue light, and therefore, it can reduce the monodehydroascorbic acid produced to the same levels as in control samples.

Despite the evidence in the scientific literature regarding the elicitor effect of blue wavelengths on the biosynthesis of ascorbic acid in several plant species [23,63,64], the underlying molecular mechanisms of this phenomenon have not yet been clarified in leaves. One aspect that warrants further investigation is the possible role of the modified blue light regime in generating reactive oxygen species (ROS) that may activate the plant’s scavenging mechanisms and subsequently stimulate the biosynthesis of antioxidants, resulting in an indirect effect of blue light on ascorbic acid accumulation [65]. Although the experimental data presented in this study do not conclusively demonstrate this assumption, the elevated levels of APX recorded suggest the onset of oxidative stress in our plants, partially supporting this hypothesis

The significant increase in malic acid and the decrease in citric acid in the CBL group might indicate an imbalance within the tricarboxylic acid cycle during the blue irradiation phase of the treatments. The rise in malic acid could, in this case, be an indicator of greater stomatal opening, in line with the observed higher nutrient solution consumption in the CBL treatments. It is known that malate is used to counterbalance potassium charges, and this influences stomatal opening [66]. Furthermore, blue light has a direct effect on promoting stomatal opening [67], and this correlates with the synthesis and translocation of malate in guard cells [68]. In our case, although we were unable to ascertain the difference between the guard cells and the rest of the leaf tissue, the induction of stomatal opening due to blue light during the night phase may have stimulated the biosynthesis of malate at the expense of citrate accumulation, for which the substrate from glycolysis might be not available due to the low photosynthetic efficiency of the irradiation. Nevertheless, higher consumption of the nutrient solution in the treatment did not lead to an elevation in nitrates, sulfates, or phosphates that could compromise the product quality if they exceed certain concentration limits.

## 5. Conclusions

In conclusion, our experiments propose an efficient and simple method to improve the content of AsA in *Eruca vesicaria* grown under fully controlled conditions. Given the importance of this vitamin in human nutrition, the use of continuous blue light proves to be a promising strategy to enhance the quality of vegetables in CEA with no detrimental effect on productivity or other quality variables. Nevertheless, while quantifying the benefits of light use for quality improvement, it is essential to take into account the potential drawback of increased water consumption associated with the impact of continuous blue light and verify that the physiological response to blue light, as we used it, is shared by other relevant species in addition to *Eruca vesicaria*.

The analysis of enzyme activity indicated that the increase in the ascorbic acid pool observed in the CBL treatments is due to the activity of the main biosynthetic pathway rather than that of the ascorbic acid recycling system. However, the data provided in this work and in the scientific literature are still insufficient to fully understand the physiological mechanism underlying the interaction between blue light and ascorbic acid biosynthesis in leafy crops. Future research on the physiological mechanisms involved, including a detailed study of the gas exchange, time-related dynamics of the ascorbic acid increase, and molecular analyses could elucidate the role of blue light and CBL treatments in the biosynthesis of ascorbic acid.

## Figures and Tables

**Table 1:** Mean comparisons for growth-related traits of fresh weight, dry weight, specific leaf dry weight (SLDW), ash, and leaf area index (LAI) recorded at 20 days after time zero of *Eruca vesicaria* L. grown under CBL and Control conditions.

	Fresh Weight	Dry Weight	Dry Matter	SLDW	Ash	LAI
	(g/m^2^)	(g/m^2^)	(%)	(g/m^2^)	(% DW)	
CBL	3471.06 ± 302	239.17 ± 22.2	6.86 ± 0.09	40.00 ± 2.6	7.07 ± 0.39	7.43 ± 0.70
Control	3111.47 ± 208	193.51 ± 11.9	6.24 ± 0.05	33.36 ± 1.3	7.80 ± 1.16	6.53 ± 0.59
Sign.	ns	ns	***	*	ns	ns

*, *** Significant for *p* < 0.05 and 0.001, ns = not significant.

**Table 2:** Mean comparison for non-structural carbohydrates including glucose (Glu), fructose (Fru), sucrose (Suc), starch, total soluble carbohydrates (Tot. sol.), and total non-structural carbohydrates (Tot. NSC) of *Eruca vesicaria* L. grown under CBL and Control.

	Glu	Fru	Suc	Starch	Tot. sol	Tot. NSC
	(% DW)					
CBL	12.48 ± 2.6	1.85 ± 0.52	7.65 ± 0.43	5.56 ± 0.38	21.98 ± 3.4	27.53 ± 3.7
Control	10.45 ± 2.9	1.52 ± 0.64	6.73 ± 0.51	5.83 ± 0.32	18.70 ± 3.6	24.53 ± 3.8
Sign.	ns	ns	ns	ns	ns	ns

ns = not significant.

**Table 3:** Mean comparison for pigment content including chlorophyll *a* (Chl *a*), chlorophyll *b* (Chl *b*), total chlorophylls (Tot. Chl), chlorophylls ratio (Chl*a*/Chl*b*), β- carotene, lutein (Lut), neoxanthin (Neo), violaxanthin (Vio), total anthocyanin (Tot. Ant), and total phenolic content (TPC) of *Eruca vesicaria* L. grown under CBL and Control conditions.

	Chl *a*	Chl *b*	Tot. Chl	Chl*a*/Chl*b*	β-Car	Lut	Neo	Vio	Tot. Ant	TPC
(mg 100 g^−1^ FW)										
CBL	93.0 ± 5.0	33.8 ± 1.9	126.9 ± 7.0	2.7 ± 0.16	6.6 ± 0.66	9.0 ± 0.44	3.0 ± 0.24	102.4 ± 7.3	0.89 ± 0.08	105 ± 7.9
Control	77.9 ± 3.0	28.6 ± 1.1	106.5 ± 4.1	2.7 ± 0.16	6.0 ± 0.24	7.6 ± 0.33	2.6 ± 0.13	93.7 ± 6.6	0.77 ± 0.05	88.2 ± 4.3
Sign.	*	*	*	ns	ns	*	ns	ns	ns	ns

* Significant for *p* < 0.05, ns = not significant.

**Table 4:** Mean comparison for anion content including nitrate, sulfate, phosphate, malate, and citrate of *Eruca vesicaria* L. grown under CBL and Control.

	Nitrate	Sulfate	Phosphate	Malate	Citrate
	(ppm)	(ppm)	(ppm)	(mg 100 g FW^−1^)	(mg 100 g FW^−1^)
CBL	4929.7 ± 293.1	1010.6 ± 74.2	1047.4 ± 74.3	470.6 ± 47.5	71.3 ± 8.7
Control	4502.0 ± 247.4	1102.2 ± 77.0	1316.8 ± 325.8	228.0 ± 16.9	124.2 ± 19.1
Sign.	ns	ns	ns	***	*

*, *** Significant for *p* < 0.05 and 0.001, ns = not significant.

## Data Availability

The original contributions presented in the study are included in the article, further inquiries can be directed to the corresponding author.
